# Twenty years’ experience of primary vaginal cancer treatment at one cancer centre: does residence status matter?

**DOI:** 10.3332/ecancer.2021.1267

**Published:** 2021-07-15

**Authors:** Olga P Matylevich, Hanna V Trukhan, Olga I Zubets, Siarhei A. Mavrichev

**Affiliations:** 1 Gynecologic Oncology Department, NN Alexandrov National Cancer Centre of Belarus, a/g Lesnoy-2, 223040 Minsk, Belarus; 2 Department of Oncology, Belarusian Medical Academy of Postgraduate Education, Minsk, Belarus, Brovki Street, 3, build. 3, 220013 Minsk, Belarus; 3 Cancer Control Department, NN Alexandrov National Cancer Centre of Belarus, a/g Lesnoy-2, 223040 Minsk, Belarus

**Keywords:** vaginal cancer, long-term results, urban / rural patients, differences in diagnosis and survival

## Abstract

**Objectives:**

To study the long-term results of the treatment of patients with vaginal cancer and to examine whether there are any differences in diagnostic and survival rates between urban and rural patients.

**Methods:**

The data of 70 patients with primary vaginal cancer treated at NN Alexandrov National Cancer Centre of Belarus from 2000 to 2019 were included. The median age was 64 years (range = 56–75). Morphology in 91.4% (64/70) of the cases was squamous cell cancer, in 7.1% (5/70) it was adenocarcinoma and in 1.4% (1/70) it was adenosquamous carcinoma. In total, there were 31 patients from urban and 39 from rural areas. The groups were comparable in age (61 versus 67, *p* = 0.104), morphology (*p* = 0.188) and distribution of stages: stage I in 7 and 10 patients (22.6% and 25.6%, respectively; *p* = 0.999), stage II in 14 and 16 patients (45.1% and 41.0%, respectively; *p* = 0.810), stage III in 6 and 6 patients (19.4% and 15.4%, respectively; *p* = 0.754) and stage IV in 4 and 7 patients (12.9% and 18.0%, respectively; *p* = 0.744).

**Results:**

The median follow-up time was 33 months (range = 1–220). A total of 42 women died: 28 from progression of vaginal cancer and 14 from other diseases. Overall survival (OS) was 31.9 ± 6.8%, median survival was 41 months (95% CI = 0.0–105.3). Disease-specific survival (DSS) for the entire group was 54.5 ± 6.8%; median was not reached. The overall survival rate of urban women was 44.8 ± 10.6% and for rural it was 22.5 ± 8.2% (*p* = 0.142); DSS was 57.6 ± 10.5% and 53.0 ± 8.4% (*p* = 0.448), respectively.

**Conclusion:**

DSS rate was 54.0 ± 6.8% and the OS rate did not exceed 31.9 ± 6.8%. Rural residence was not associated with late stage at diagnosis or receipt of treatment.

## Introduction

Vaginal cancer is a rather rare pathology and accounts for only 1%–2% of all the cases of cancer of the female reproductive system; it most often occurs at the age of 60–70 years [1–3]. In the United States, 8,180 new cases of vaginal cancer are diagnosed annually and 1,530 patients die [4]. In the Republic of Belarus, about 35 new cases of vaginal cancer are detected annually and 12 patients die from this disease [5]. According to the literature, about 70% of all the cases of vaginal cancer are associated with HPV and are characterised by many of the same risk factors as cervical cancer: multiple sexual partners, early age at first intercourse and smoking [6–8]. The choice of treatment for patients with vaginal carcinoma depends on a number of factors, such as the stage of the disease, the anatomical location of the lesion, the histological structure of the tumour, the volume of the tumour and the age of the patient. In the case of vaginal cancer, a variety of treatments may be offered, including surgery, radiation therapy, chemotherapy or combinations thereof [2–4, 9].

There is evidence from some authors on the existence of differences in diagnosis and survival rates for cervical cancer, lung cancer, colorectal and other types of cancer between urban and rural residents due to health inequalities associated with cancer [10–13]. In Belarus, schemes for the diagnosis and treatment of vaginal cancer in urban and rural patients have not been studied.

The aim of this study was to summarise the experience of one cancer centre in treating vaginal cancer over a 20-year period and to evaluate the results of diagnosis and treatment of patients living in urban and rural areas.

## Methods

### Patient selection

The retrospective cohort study included data from 104 patients with vaginal malignant neoplasms (C52), whose treatment was carried out at NN Alexandrov National Cancer Centre of Belarus for the period from 2000 to 2019. Twenty-five women with multiple primary tumours and nine women with non-epithelial vaginal tumours (melanoma, sarcoma etc.) were excluded from the study.

Seventy patients with primary vaginal cancer met the inclusion criteria. All tumours were confirmed histologically. All International Federation of Gynaecology and Obstetrics (FIGO) 2009 [3] stages I–IV and the following histology were included: squamous cell carcinoma, adenocarcinoma and adenosquamous carcinoma. Patient demographics, pathological data and follow-up information were retrospectively collected from electronic medical records. This study was approved by the Ethics Committee of the NN Alexandrov National Cancer Centre (protocol No17), Minsk, Belarus, on 17 January 2020.

### Treatment

Treatment was carried out in the vast majority of patients – in 81.4% (57/70) of the cases: with stage I of the disease in 94.1% (16/17), with stage II in 83.3% (25/30) of the cases, with stage III in 91.7% (11/12) of the cases and with stage IV in 45.5% (5/11) of the cases. In 13 patients, treatment was not carried out due to the refusal of patients at stages I and II, as well as because of an incurable condition at stage IV.

At stage I of the disease, radiation therapy was most often used – a combination of external beam radiation therapy with intracavitary brachytherapy and/or interstitial brachytherapy – in 10 (62.5%) patients and 6 (37.5%) patients with upper vaginal disease underwent surgery, after which 2 patients received an adjuvant course of radiotherapy. At stage II, 16 (64.0%) patients underwent a course of combined radiation therapy (external beam radiotherapy and brachytherapy), 4 (16.0%) underwent a course of external radiation therapy with lack of conditions for brachytherapy, 5 (20.0%) underwent surgery and 1 underwent a postoperative course of external beam radiation therapy. All (100%) stage III patients underwent a course of combined chemoradiation therapy or external beam chemoradiation therapy. At stage IV of the disease, palliative radiation with chemotherapy was used.

### Statistical analysis

Data were summarised using basic descriptive statistics. The main outcome of the study *was survival time. The time period was calculated at the time* of diagnosis. Survival rates were calculated according to Kaplan–Meier for the entire observation period. Comparison of survival in two groups was carried out according to the Log-rank test, and in groups of three or more using the *c*^2^ criterion. Differences were considered statistically significant at p <0.05. All *p*-values were two-sided. The calculations were carried out using the Statistical Package for the Social Sciences (IBM SPSS Statistics for Windows version 23.0, Armonk, NY, USA).

## Results

### Cohort characteristics

We identified 70 patients with primary vaginal cancer. Patient and tumour characteristics are detailed in Table 1.

The median age was 64 years (range = 56–75). Morphology in 91.5% (64/70) of the cases was squamous cell cancer, in 7.1% (5/70) it was adenocarcinoma and in 1.4% (1/70) it was adenosquamous carcinoma. The distribution by the stages was as follows: stage I in 17 (24.3%) patients, stage II in 30 (42.9%) patients, stage III in 12 (17.1%) patients and stage IV in 11 (15.7%) patients.

**Figure 1.**
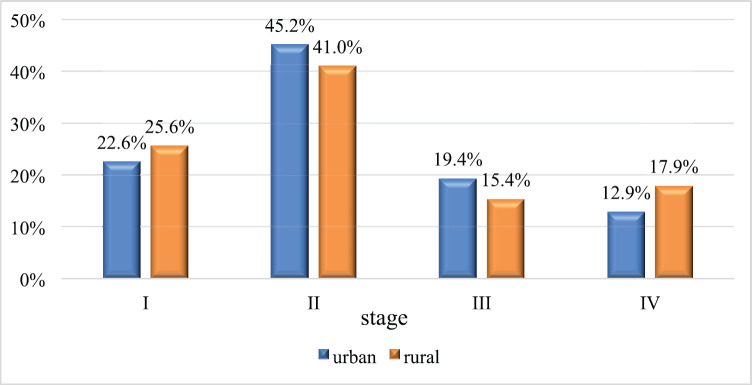
Vaginal cancer case distribution in urban and rural areas by stages of the disease.

In total, there were 31 patients from the city and 39 from the countryside. The groups were comparable for the following clinical and morphological characteristics: age (*p* = 0.104) – the age of urban women varied from 32 to 87 years, the median was 61 years, and the age of rural women varied from 51 to 73 years, the median was 67 years; morphological structure (*p* = 0.188); and stages of the disease: stage I in 7 and 10 patients (22.6% and 25.6%, respectively; *p* = 0.999), stage II in 14 and 16 patients (45.2% and 41.0%,respectively; *p* = 0.810), stage III in 6 and 6 patients (19.4% and 15.4%, respectively; *p* = 0.754) and stage IV in 4 and 7 patients (12.9% and 17.9%, respectively; *p* = 0.744) (Figure 1).

### Survival outcomes

**Figure 2.**
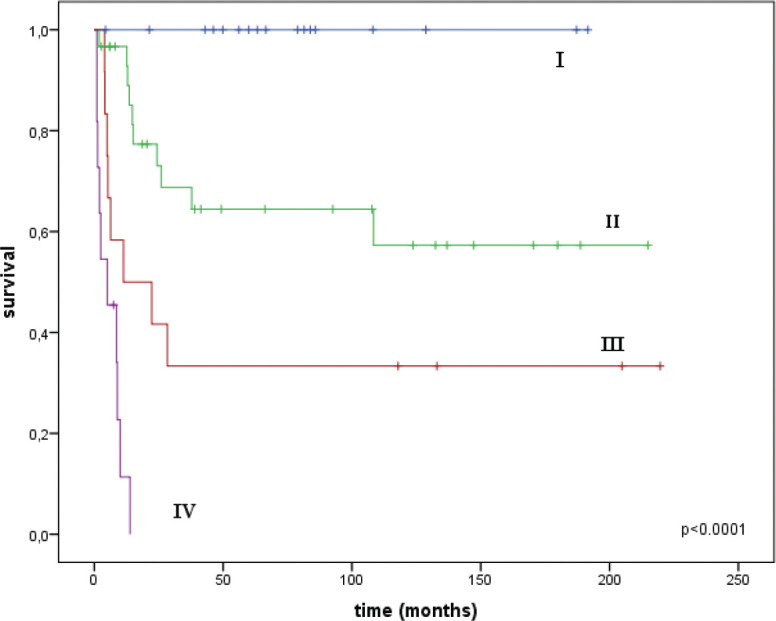
DSS curves for the entire group by stages.

The median follow-up time was 33 months (range = 1–220). A total of 42 women died: 28 from the progression of vaginal cancer and 14 from other diseases. The overall survival (OS) rate was 31.9 ± 6.8% and median survival was 41 months (95% CI = 0.0–105.3). The disease-specific survival (DSS) rate for the entire group was 54.5 ± 6.8%; median was not reached. The DSS by stages was as follows: at I stage it was 100%, at stage II it was 57.3 ± 10.9%, at stage III it was 33.3 ± 13.6%, no patients with stage IV survived a year and 1-year survival was 11.4 ± 10.5% (Figure 2).

With a median follow-up of 13 months (range = 1–23), seven (10%) patients had recurrence and all patients died. In the remaining, the cumulative eventless survival rate after the diagnosis was 85.3 ± 5.3%.

### Subgroup analysis: urban versus rural

**Figure 3.**
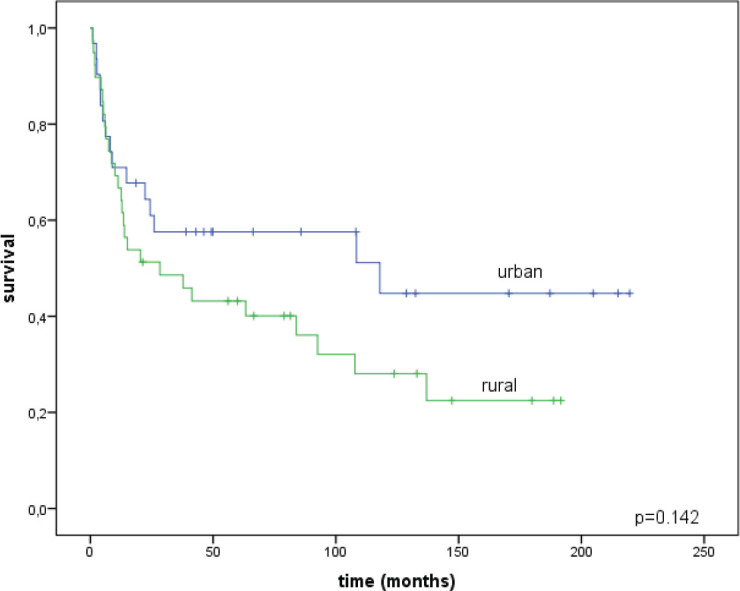
Overall survival rates for urban and rural patients.

The median follow-up time was 43 months for urban patients (range = 1–220) and 21.4 months for rural patients (range = 1–192; *p* = 0.449). We compared the survival outcomes between urban and rural groups. The overall survival rate of urban women was 44.8 ± 10.6% and for rural women it was 22.5 ± 8.2% (*p* = 0.142), without statistically significant differences (Figure 3).

**Figure 4.**
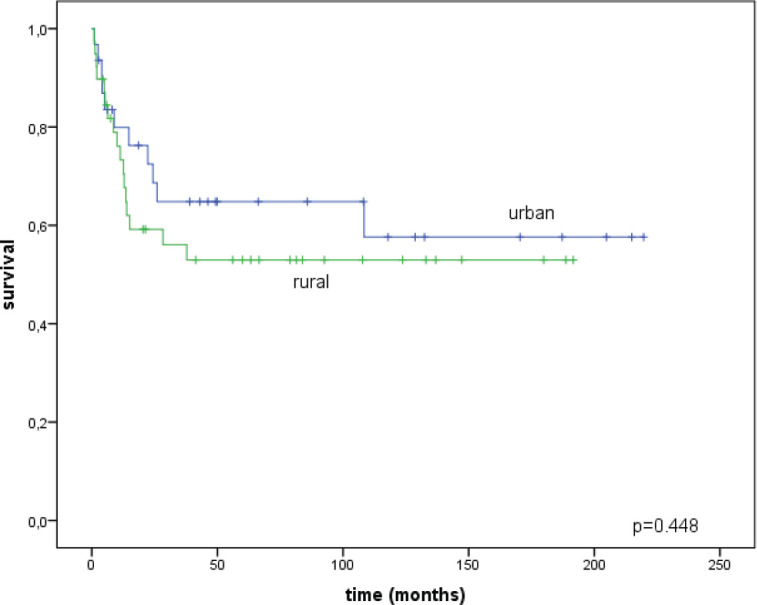
Disease-specific survival curves for urban/rural residents.

The disease-specific survival was also similar between the two groups: for women in the city it was 57.6 ± 10.5% and for rural women it was 53.0 ± 8.4% (*p* = 0.448) (Figure 4).

**Figure 5.**
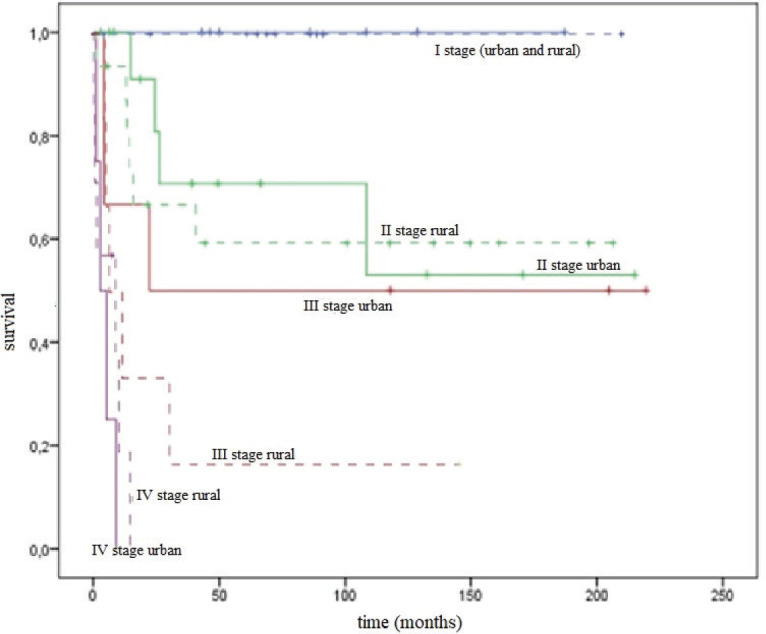
DSS curves for urban/rural residents by stages.

The DSS rate of urban and rural residents by stages was comparable: for stage I it was 100% in both groups; for stage II it was 53.0 ± 18.7% and 59.5 ± 12.9%, respectively (*p* = 0.670); and for stage III it was 50.0 ± 20.4% and 16.7 ± 15.2%, respectively (*p* = 0.415). Patients with stage IV in the group of urban women died within 5 months and rural women within 1 year (Figure 5).

## Discussion

Primary vaginal cancer is rare. Historically, these cancers are more common in older women and postmenopausal women [3]. The median age of our patient population was 64 years, which confirms the literature data.

Since most vaginal malignancies have squamous cell histology, the aetiology is common with cervical cancer. This is the persistence of high-risk oncogenic HPV infections. According to our data, the predominant tumour structure was also squamous cell carcinoma (91.5%), other morphological variants were extremely rare.

Because vaginal cancer is uncommon, in the published literature there is much debate and controversy over the preferred treatment. Most of the management is extrapolated from cervical cancer due to similar aetiology and anatomical location. With early diagnosis, both surgical resection and radiation therapy can be curative options. In most patients, especially in advanced stages, radiation, consisting of external beam radiation and brachytherapy, plays a central role. Accordingly, the outcomes are influenced by the experience of the medical team in treating this unique tumour [14–16]. In our study, majority of the patients received anti-tumour treatment (82.9%). The most frequent method of treatment at stages I–III was a definitive radiation therapy: in 62.5%, 80% and 100% of the cases, respectively. The DSS rate was 54.0 ± 6.7% and the overall survival rate did not exceed 31.6 ± 6.7%. The DSS by stages was as follows: at stage I stage it was 100%, at stage II it was 57.3±10.9%, at stage III it was 33.3 ±13.6%, none of the patients with stage IV survived for a year and 1-year survival rate was 11.4 ± 10.5%. Relatively, 5-year survival in a larger series ranged from 64 to 84% for stage I, 53–58% for stage II, 36% for stage III and 18%–36% for stage IV [17]. Our data are consistent with the current literature rates, with the exception of stage IV of the disease [14–18]. The low survival rate of patients with advanced stage of vaginal cancer is attributed to the lack of effective treatment for this category of patients in our setting. Thus, given the limited options for patients with recurrent and metastatic cervical cancer, which is similar in nature to vaginal cancer, Kahn et al [19] recommend considering the possibility of immunotherapy with pembrolizumab in PD-L1-positive patients.

Few studies have examined the role of residence in diagnosis and treatment outcomes in patients with cervical cancer, which is the closest in aetiology to vaginal cancer and other cancers [10–13]. Singh [10] reports that in the United States the 5-year survival rate for black women diagnosed with cervical cancer was 50.8% in the non-metropolitan areas, compared with 60.2% for black women and 71.0% for white women in metropolitan areas. Differences in survival were observed after taking into account the stage of the disease. Urban–rural disparities in cervical cancer incidence persist, despite sharp declines in morbidity and mortality. In our study, if we analyse the role of the place of residence, there are no differences in stage distribution between urban and rural groups. If we look at the survival outcomes, there are no discrepancies in overall, disease-specific survival and survival by the stages between women living in urban and rural areas. In our opinion, this is due to the presence of centralised oncological care in Belarus and the provision of the healthcare system with universal access to medical care, which is free at the point of use [20].

This study is limited by its retrospective cohort analysis with a small sample size and being a single cancer centre study with possible referral bias. However, survival of patients in present report was compared to the literature data. Both OS and DSS are consistent with current literature estimates [14–18].

## Conclusion

Because primary vaginal cancer is rare, treatment is complex and often individualised. Therefore, it is recommended, whenever possible, to refer women diagnosed with primary vaginal cancer to specialised cancer centres. In our study, there were no differences in the stages of diagnosis and treatment outcomes between urban and rural patients, which indicate the same access to diagnostic and medical services.

## Conflicts of interest

The authors declare that they have no conflicts of interest.

## Authors’ contribution

OPM carried out data collection, analysis and wrote the manuscript. HVT and SAM were involved in the treatment of patients, analysis and manuscript editing. OIZ carried out the statistical analysis. All authors approved the final version of the manuscript.

## Figures and Tables

**Table 1.** Patient and tumour characteristics.

Category	Total (*n* = 70)	Urban (*n* = 31)	Rural (*n* = 39)	*p*-value		
Age (years), median (range)	64 (56–75)	61(32–87)	67 (51–73)	0.104		
**Histology, *n* (%)**						
Squamous	64 (91.4%)	27 (87.1%)	37 (94.9%)	0.395		0.188
Adenocarcinoma	5 (7.1%)	3 (9.7%)	2 (5.1%)	0.649		
Adenosquamous	1 (1.4%)	1 (3.2%)	0	0.443		
**FIGO (2009) Stage, *n* (%)**						
I	17 (24.3%)	7 (22.6%)	10 (25.6%)	0.999		0.985
II	30 (42.9%)	14 (45.1%)	16 (41.0%)	0.810		
III	12 (17.1%)	6 (19.4%)	6 (15.4%)	0.754		
IV	11 (15.7%)	4 (12.9%)	7 (18.0%)	0.744		
**Treatment**						
Yes	57 (81.4%)	27 (87.1%)	30 (76.9%)	0.440		0.280
No	13 (18.6%)	4 (12.9%)	9 (23.1%)	0.440		
**Relapses, *n* (%)**						
Yes	7	2 (6.5%)	5 (12.8%)	0.452	0.381	
No	63	29 (93.5%)	34 (87.2%)	0.452		
**Follow-up (months), median (range)**	33 (1–219.6)	43 (1–219.6)	21.4 (1–191.6)	0.449		

Abbreviations: FIGO, International Federation of Gynaecology and Obstetrics

## References

[1] Siegel RL, Miller KD, Jemal A (2016). Cancer statistics, 2016. CA Cancer J Clin.

[2] Eifel P, Klopp AH, Berek JS, DeVita VT, Lawrence TS, Rosenberg SA (2019). Chapter 74: cancer of the cervix, vagina, and vulva. DeVita, Hellman, and Rosenberg’s Cancer: Principles and Practice of Oncology.

[3] Adams TS, Cuello MA (2018). Cancer of the vagina. Int J Gynaecol Obstet.

[4] American Cancer Society (2021). Cancer Facts & Figures 2021.

[5] Okeanov AE, Sukonko OG (2018). Statistics of Oncological Diseases in the Republic of Belarus (2008–2017).

[6] Sinno AK, Saraiya M, Thompson TD (2014). Human papillomavirus genotype prevalence in invasive vaginal cancer from a registry-based population. Obstet Gynecol.

[7] Larsson GL, Helenius G, Andersson S (2013). Prognostic impact of human papilloma virus (HPV) genotyping and HPV-16 subtyping in vaginal carcinoma. Gynecol Oncol.

[8] Daling JR, Madeleine MM, Schwartz SM (2002). A population-based study of squamous cell vaginal cancer: HPV and cofactors. Gynecol Oncol.

[9] https://www2.tri-kobe.org/nccn/guideline/gynecological/english/vulvar.pdf.

[10] Singh GK (2012). Rural-urban trends and patterns in cervical cancer mortality, incidence, stage, and survival in the United States, 1950–2008. J Community Health.

[11] Johnson AM, Hines RB, Johnson JA (2014). Treatment and survival disparities in lung cancer: the effect of social environment and place of residence. Lung Cancer.

[12] Hines R, Markossian T, Johnson A (2014). Geographic residency status and census tract socioeconomic status as determinants of colorectal cancer outcomes. Am J Public Health.

[13] Markossian TW, Hines RB (2012). Disparities in late stage diagnosis, treatment, and breast cancer-related death by race, age, and rural residence among women in Georgia. Women Health.

[14] Gardner CS, Sunil J, Klopp AH (2015). Primary vaginal cancer: role of MRI in diagnosis, staging and treatment. Br J Radiol.

[15] Frank SJ, Jhingran A, Levenback C (2005). Definitive radiation therapy for squamous cell carcinoma of the vagina. Int J Radiat Oncol Biol Phys.

[16] Creasman WT (2005). Vaginal cancers. Curr Opin Obstet Gynecol.

[17] de Crevoisier R, Sanfilippo N, Gerbaulet A (2007). Exclusive radiotherapy for primary squamous cell carcinoma of the vagina. Radiother Oncol.

[18] Pingley S, Shrivastava SK, Sarin R (2000). Primary carcinoma of the vagina: tata memorial hospital experience. Int J Radiat Oncol Biol Phys.

[19] Kahn RM, Gordhandas S, Craig K (2020). Cervical carcinoma in the setting of uterovaginal prolapse: comparing standard versus tailored management. Ecancermedicalscience.

[20] Richardson E, Malakhova I, Novik I (2013). Belarus: health system review. Health Syst Transit.

